# Barriers and enablers to opioid deprescription: A qualitative study

**DOI:** 10.1371/journal.pone.0316730

**Published:** 2025-01-07

**Authors:** Rebecca Lawrence, Everett Versteeg, Andrea Pike, Holly Etchegary, Amanda Hall

**Affiliations:** 1 Primary Healthcare Research Unit, Memorial University, St. John’s, Newfoundland and Labrador, Canada; 2 Pasqua Hospital, Regina, Saskatchewan, Canada; 3 Division of Community Health and Humanities, Memorial University, St. John’s, Newfoundland and Labrador, Canada; PLOS: Public Library of Science, UNITED KINGDOM OF GREAT BRITAIN AND NORTHERN IRELAND

## Abstract

**Background:**

Canada has the fourth highest per capita rate of opioid prescriptions in the world, contributing to the country’s opioid crisis. Due to both their pain-relieving and euphoric properties, opioids can be highly addictive, leading to potential overdose and death. Deprescription is an endorsed and organized method of discontinuing a drug but very little is known about the barriers that Canadian physicians face when attempting to deprescribe opioids, particularly those who practice in rural areas (which have some of the highest rates of opioid users).

**Methods:**

This was an explorative, qualitative study describing rural family doctors’ experiences and practices regarding opioid deprescription in primary care. A convenience sample of family doctors who had experience working with patients taking opioid medications was recruited from the professional networks of study team members. After consenting to participate, data was collected using semi-structured telephone interviews and analyzed by researchers experienced in applying the Theoretical Domains Framework to assess barriers and enablers of behavior change.

**Principal findings:**

10 physicians participated in this study. Our analysis revealed four barriers and five enablers related to opioid deprescription in rural primary care. Barriers include a lack of knowledge and skills related to deprescribing, discomfort initiating deprescription, patient pressure to continue prescribing opioids, and a lack of foundational support required to deprescribe. Enablers include working with colleagues who share common views on overuse of opioids and deprescription; access to other healthcare providers, community-based resources, and clinical tools; using a systematic approach to deprescription; previous experience successfully deprescribing opioids; and practicing in a rural setting.

**Conclusions:**

Opioid dependence and over-prescription continue to be a problem for our health system. Deprescription is necessary but challenging for family physicians. Rural physicians are keenly aware of the importance of preserving the physician-patient therapeutic relationship and open and clear communication about opioid medications and deprescription but feel unprepared to manage this in the face of difficult issues surrounding deprescription. They also feel unprepared to deal with deprescription effectively without access to other resources, healthcare professionals, patient education materials and time. Rural physicians would benefit most from added foundational supports for deprescription.

## Introduction

Opioids are a class of pain-relieving drugs often prescribed to reduce pain and improve functioning [1]. Opioids work by attaching to opioid receptors in the brain, which results in an alteration of the perception of pain by the nervous system [1]. However, opioids also produce a sense of euphoria and well-being which can be addictive [1,2]. For patients with chronic non-cancer pain (CNCP), current guidelines suggest that opioids should be reserved for those who have failed to respond to non-opioid medications or other first-line treatments [3]. Despite these guidelines, between the years 2013 to 2018, only one in four patients who were prescribed opioids were trialled on a non-opioid medication before commencing opioid therapy [4]. Choosing Wisely Canada (CWC) has also published recommendations for the use of opioids for various medical specialities. They suggest that opioids should not be continued beyond immediate post-operative care or other episodes of acute severe pain in the primary care setting. In these instances, prescriptions should be no longer than seven days [5]. Similarly, CWC recommends that opioids should not be initiated for CNCP as current evidence suggests that opioids are not useful to treat chronic pain conditions such as chronic low-back pain, fibromyalgia, arthritis, or recurrent headaches [5,6].

Canada has the fourth highest per capita rate of opioid prescriptions in the world, contributing to the country’s opioid crisis [1,7]. A study evaluating Canadian prescribing practices found that one in eight individuals in the study population were prescribed opioids in the year 2018 [4]. Due to both their pain-relieving and euphoric properties, opioids can be highly addictive, leading to potential overdose and death [6]. Between January 2016 and December 2021, Canada reported 29,052 apparent opioid toxicity deaths [8], reflecting the dangers of misusing and inappropriately prescribing opioids. Indeed, more than 600,000 people have died from apparent opioid toxicity since 1999 and the opioid overdose mortality rate in each country is greater that the mortality rate at the height of the HIV/AIDS epidemic [9–13].

Prescribing patterns have been changing in recent years [4]. Between the years 2013 and 2018, the number of opioid prescriptions decreased by 8% [4]. Similarly, during this period, the number of patients who began taking opioids and who were prescribed opioids long-term decreased [4]. Despite this headway, pharmaceutical opioids (those prescribed by a physician) still accounted for approximately 20% of opioid-related deaths in 2023 [8]. This highlights the importance of continued efforts toward improving guideline-concordant prescribing of opioid medications.

Deprescription is an organized method of discontinuing a drug [14] and has been endorsed by organizations such as the National Advisory for Prescription Drug Misuse [15]. A systematic review of qualitative studies worldwide found that one of the barriers to deprescribing opioids was both the lack of, and access to, appropriate alternatives such as chronic pain clinics [16]. The average wait time for a publicly funded chronic pain clinic in Canada is six months; in some provinces, this wait can be up to five years [17]. Other reported barriers included a lack of patient understanding of the risks of continued opioid use, gaps in provider training, lack of time, and fear of damaging the patient-provider relationship [16].

Very little is known about the barriers that Canadian physicians face when attempting to deprescribe opioids, and more specifically Canadian physicians who practice in rural areas. Recent evidence has shown that rural areas have some of the highest numbers of prescription opioid users [18]. This is notably important for the Canadian province of Newfoundland and Labrador (NL) where nearly 40% of the population resides in rural communities [19]. Therefore, the purpose of this study is to better understand the barriers and enablers faced by family physicians practicing in rural NL when deprescribing opioids for CNCP.

## Methods

This was an explorative, qualitative study describing rural family doctors’ experiences and practices regarding opioid deprescription in primary care. Ethics approval for this study was obtained from the Health Research Ethics Board in Newfoundland and Labrador (HREB 20180366).

### Participants

Eligible participants included family doctors with at least one year of active practice in rural NL who had experience working with patients taking opioid medications. Statistics Canada defines rural areas as those that are outside the commuting zone of larger urban centers, those with populations exceeding 100,000 people [20]. Therefore, all areas outside of the commuting zone of St. John’s were considered rural for the purpose of this study. We attempted to identify participants by sending an email to the Newfoundland and Labrador College of Family Physicians of Canada list, soliciting physicians working in rural NL to participate. When that strategy failed, members of the study team (EV & AH) leveraged their networks of physician colleagues, reaching out directly to inform them about the study and invite them to participate. The recruitment period, beginning with our initial recruitment strategy ran from August 23, 2017 to August 20, 2020. Healthcare services in NL are publicly funded and delivered through a single health authority with four health zones: the eastern zone is the largest and services approximately half the population, while the central, western, and Labrador-Grenfell zones service more rural catchment areas. Purposive sampling [21] was used to ensure we recruited participants from each of the four provincial health zones so that any variation in staff, regulations, or training could be captured in the interviews. Following the sample size recommendations for qualitative, theory-based interview studies proposed by Francis et al. [22] we planned to recruit a minimum of 13 family doctors for this study (initial analysis sample of 10 + a stopping criterion of three interviews), completing additional sets of three interviews as required until data saturation was reached (no new themes emerging after three consecutive interviews). No participants refused participation or dropped out.

### Data collection

Data was collected using semi-structured telephone interviews. Potential participants were invited by email to take part in a study investigating the barriers and enablers to deprescribing opioids for patients with CNCP. Interested participants were provided with additional information about the study and emailed the study information form disclosing all considerations of the project and their rights as a participant. Interviews were then scheduled for a mutually convenient time for the participant and researchers. Oral consent to participate was provided to the interviewer orally prior to beginning the interview. The interview guide (please see S1 Appendix) included a total of five questions. Additional questions and prompts were included during the interviews to help interviewers clarify participants’ responses.

Interviews were conducted by one of three female project team members (AH and MVW who are PhD and Master’s-prepared or CM a medical student). All three had basic qualitative training with two (AH, MVW) having more extensive experience with qualitative interviewing. Interview respondents were not previously known to the interviewers. All interviewers were employed by Memorial University at the time the interviews were completed; none of the interviewers were prescribers of opioids or have a vested interest in any deprescribing programs. Interviewers explained the purpose of the study and provided their credentials but did not disclose any other information about themselves to interview participants. All interviews were conducted over the phone; consent was provided orally prior to beginning the interview. Participants were provided a $50 honorarium for their participation.

The interviews took approximately 30 minutes to complete and were audio-recorded and transcribed verbatim. Field notes were taken by the interviewers (as a back up in case of recording problems) but not used in the analysis. Participants were not provided the opportunity to review their transcripts and no repeat interviews were conducted.

### Data analysis

We used the Atkins et al [23] guide for applying the Theoretical Domains Framework (TDF) to assess barriers and enablers of behavior change to analyse our data. The TDF is a framework designed to identify influences on health professionals’ behaviour related to the implementation of evidence-based guidelines and recommendations. Originally developed by Michie et al. [24], the current version of the TDF [25] is a synthesis of 84 key theoretical constructs into 14 overarching domains (e.g., social influences, knowledge, environmental context and resources) presented in a single framework. Using the TDF to generate a framework for content analysis, researchers analysed the data deductively (by assigning text to one or more domains) and inductively (generating themes at each of the domains).

#### Deductive analysis

Two researchers (MVW & RL) were trained to code the transcripts using the TDF coding scheme as described by Atkins et al. [23] using Microsoft Word and NVivo. The coders first read and reread the transcripts to become familiar with the data and ensure that the interviews were of similar quality and depth before proceeding to code the first interview together. Coding of the interview was finalized when they reached consensus on all coded passages. The second interview was coded independently then reviewed together to reach consensus. Subsequent interviews were jointly reviewed after every second transcript was coded to promote consistency.

#### Inductive analysis

After the interview transcripts were coded into the TDF domains, themes were identified among the data coded under each domain, phrased as belief statements about barriers and enablers to opioid deprescription. These were further refined to reflect broader themes or patterns in the data. All belief statements and broad themes (with supporting quotes) were reviewed by two senior researchers (AP & AH).

## Results

A total of 10 family physicians practicing in rural areas of each of NL’s health zones participated in this study (two each from the Eastern, Central, and Western health zones and four from the Labrador-Grenfell zone). Participants had a range of experience from one to 10 years in rural practice. While additional interviews were planned to formally assess data saturation, the research team made the decision to stop recruitment at 10 interviews due to resource constraints and the overall burden of the Covid-19 pandemic on family doctors. Recruitment during pandemic was challenging and it took us much longer than anticipated. Instead, we decided to complete an informal assessment of data saturation using the existing sample of 10 interviews. After reviewing the data and its subsequent analysis, the research team felt the data saturation requirement had been satisfied and that any benefit realized from seeking additional participants was overshadowed by the potential negative effects of requesting additional participation during the already burdensome Covid-19 pandemic.

Our analysis revealed four key barriers related to opioid deprescription described in detail below and presented in no particular order of significance. In our estimation, each of these areas is worthy of consideration for future intervention planning. Barriers and supporting quotations from interview participants can be found in Table 1. They were related to eight TDF domains (knowledge, skills, beliefs about capabilities, emotion, reinforcement, beliefs about consequences, social influences, and environmental context and resources).

**Table 1:** Barriers to opioid deprescription as reported by rural NL family physicians.

**Barrier 1: Physicians lack knowledge and skills related to deprescribing**		
TDF Domain	Belief statements (n)	Supporting Quotations
Knowledge	I was not educated sufficiently to competently treat pain/chronic pain during my training as a family doctor (2)	“Our education does not cover pain very well. It actually covers pain very poorly. I didn’t learn very much about pain in residency. I actually did an elective on chronic pain and addictions…some extra training. So, I feel like I learned stuff but it’s not well taught, it’s really not well taught.”—EV002
	I lack the knowledge on how to approach deprescribing and the use of alternative treatments (2)	“I feel a bit like I don’t have a good tool box for assessing patients as I am tapering them off. I don’t have a standardized approach to it, I don’t really know what to expect and how to help them overcome the hurdles they are going to run into as they are weaning off their opioids.”—EV007
	I am unaware of guidelines for deprescribing (1)	“I’m not, I’m not aware of them. I mean I feel like that there must be guidelines available…I know for methadone that there is kind of guidelines for how to sort of help people kind of taper down their methadone. So I’m assuming there must be guidelines and principles…I don’t know.”—EV009
Skills	I may not have the necessary skills to deprescribe opioids due to lack of experience treating patients who are taking opioids (1)	“I feel…kind of unprepared because I don’t have a lot of patients on opioids and we are just starting to see the front line of opioid misuse and opioid addictions.”—EV007
	I need additional skills in alternative pain therapies to help me with deprescribing (1)	“Cannabis is a source of treatment for chronic non-cancer pain in certain patients if you meet certain criteria. But like there’s no education on cannabis…[and] I would like to be able to learn how to go and do like a block of some sort, like a nerve block, but I don’t know how to do that, I have no access to that.”—EV010
Social professional role and identify	As a younger physician, patients are sometimes resistant to my efforts to deprescribe opioids (2)	“Umm, another doctor may have prescribed it for 15 years and being a new physician who’s eager to get them off of these medications, they’re very resistant to that.”—EV004
**Barrier 2: Physicians are uncomfortable initiating deprescription and fear it may do more harm than good**		
TDF Domain	Belief statements (n)	Supporting quotations
Beliefs about capabilities	It is difficult for me to deprescribe opioids (6)	“[They’re] probably the most challenging patients for me. I’d rather see anything over a patient that’s taking narcotics that I don’t feel they should be taking. And I don’t know if it’s because I just don’t believe in it or because it’s so difficult to fix. But it’s very challenging. I think that that’s also one reason why a lot of physicians do just continue to prescribe, because it’s a lot easier to just write a prescription for a narcotic then it is to wean.”—EV006 “So, that’s part of it as well you know, I don’t know how to overcome that sometimes, the patients’ unwillingness to try anything else. Patients are telling me that they have tried everything else in the past, nothing else works. So that makes it difficult.”—EV009
	I find it more difficult to deprescribe opioids for patients with more complex issues (1)	“I find the more rural we go…your practice change[s] right. So, I have a lot of people with other chronic disease, so you have diabetics, you have people umm with chronic mental health issues, a lot of social factors. It’s not just pain, sometimes you’re treating, people perceive it as pain, but a lot of that maybe you know, maybe the pain that they’re talking about is actually related to their diabetic neuropathy right. Which would be differently managed then giving them opioids first, but to them it’s just pain right. So, it all depends on your patient population, and the smaller the community you go into, a lot of time there is more social factors, financial factors, cause opioids are often a lot cheaper than [alternative pain medications such as] Cymbalta.”—EV004
Emotion	Deprescribing can make me feel anxious or stressed (2)	“I, it makes me I mean I do it because it’s important but it makes me very anxious you know to have these conversations sometimes because I know that people are going to get upset um and, they’re not going to be happy and it doesn’t make me feel good.”—EV009
	I fear that talking about deprescribing opioid medications for patients may damage the physician-patient relationship (2)	“Some of them, ah of course… can be difficult and really think that there is nothing else that will work. So it can be really hard, cause you don’t want to, I mean you want to have a good physician-patient relationship with them, and you don’t want to argue with them.”—EV005
Beliefs about consequences	Deprescribing opioids for my patients with pain may lead to patient harm (e.g., by decreasing their quality of life, causing withdrawal symptoms, pushing them toward street drugs where they won’t be managed properly) (3)	“It is really in the end just pain, but you know, you don’t want people you know, decreasing their quality of life and not being able to do things.” -EV003 “If I don’t [continue prescribing] and they’re like ‘okay this is awful’ then they may go in severe withdrawal or go on street drugs…” EV004
Reinforcement	Attempting to deprescribe opiates can damage the patient-physician therapeutic relationship and lead to the loss of the patient from my service. (3)	“He did come back twice asking for more opioids and both times I told him ‘we’ve already had this conversation, you know I’ve explained to you the reasons why I will not be prescribing these medications, and that answer is final’. And then I never saw him again.” EV009 “And honestly, after having that conversation with a lot of patients, you don’t see a lot of them back, right.” EV006
**Barrier 3: Physicians are influenced to continue prescribing opioids by patients’ concerns**		
TDF Domain	Belief statement (n)	Supporting quotations
Social influences	Patients/their family members pressure/influence my decision to continue prescribing opioids (10)	“In my experience when I tell one of my patients and we need to decrease the opioids because of this and that, they just say ‘no, it doesn’t make sense—I’m in pain I can’t do this’.” (EV001) “Honestly the biggest barrier is patient resistance. Often patients who have been taking opioids for a long time, I mean I hear over and over and over again… ‘if I don’t have it, I can’t function, I won’t be able to do anything’.” (EV009) “Some [patients] can be difficult and really think that there is nothing else that will work. So it can be really hard, because you want to have a good physician-patient relationship with them, and you don’t want to argue with them.” (EV005)
Environmental context and resources	Inheriting patients who are already on opioids makes it more difficult to deprescribe. (5)	“I would say that is the hardest part, is inheriting patients who are already on opioids. That’s, that’s the biggest challenge I think, for anyone.”—EV005 “The first thing that comes to mind would be kind of patients who are already on opioids that you weren’t involved in the initiation of the opioid with them, I and, you are kind of a new person involved in their care and you’re talking about trying to get them off the opioid, maybe their old physician is not around anymore, so I think kind of being a new person coming in and suggesting it and essentially not being involved in the initial contract when they’re initiated, um and trying to essentially remove something that the patient may perceive to be kind of a huge crutch in their life.”–EV002
**Barrier 4: Physicians lack foundational support required to deprescribe opioids**		
TDF Domain	Belief statement (n)	Supporting quotations
Environmental context and resources	It is difficult for me to deprescribe because there are not enough community-based and/or social supports and/or alternative care providers for me to refer patients to (8)	“[Patients] do not have enough support in the community…like social supports, physiotherapy, massage therapy, cognitive behavioural therapy…Even if I referred one of my patients to the pain clinic…that takes like three or four years to get in.”—EV001 “[In my community] we don’t have a lot of resources for individuals with opioid dependence or addiction. We don’t have an organized methadone or suboxone program. So that’s certainly one of the difficulties if somebody’s quite addicted or has any aberrant behaviors.”–EV007 “In small communities you don’t have the support groups and…there’s not often …seminars that are held for the public and all that stuff.–EV004
	It is difficult for me to deprescribe patients who don’t have the resources to cover costs of treatment programs and substitute medications (3)	“One challenge would be not having insurance for physiotherapy…and massage and things like that.”–EV005 “Another challenge would be drug coverage. So, here I find like opioids tend to get covered like NLPDP and stuff when it comes to opioids have like no problem, like whatever. Like you want Dilaudid you get Dilaudid, right. But when it comes to things like Lyrica, and like the other, the other non-opioid treatments, sometimes I got to fight to get them covered. So that’s a bit of an issue, and of course cost. “- EV010
	Difficulties communicating/collaborating with other healthcare providers can be a barrier to deprescribing (2)	“When you can access a resource, that resource is accessing in isolation and so I may be able to finally convince a patient to go and see a counselor but I have no idea what their counselor is telling to them, telling them, what they’re talking about or how that impacts the treatment that I’m, I’m giving them. And so, you know the patient goes and says oh my counselor says that you should give me this and… I don’t know right? I mean I don’t have communication, so we need to be working in teams… um and-and… “- EV009
	It takes more time to explain to a patient why they do not need opioids than to prescribe an opioid (1)	“Yeah, nerve pain like people don’t, they think, and then if like they explain a patients like the pathway for nerve pain, it’s like, I don’t expect if I give you dilaudid that it’s actually going to work because it’s not going to treat that type of pain. So like, it, but patients are really good when I take the time to sit down and do it but we’re talking like sometimes that conversation is like a 20–30 minute conversation so that puts me behind and then you know like you’re trying to see other people, so, yeah. So explaining to patients that’s what I find challenging to do.”—EV010
Social influences	Fear of censure from the college of physicians and surgeons is a barrier to deprescription (1)	“I think I would be more comfortable de-prescribing opioids and I think that’s one of the barriers of lots of positions just think twice before having a conflict with a patient about opioids is the college policy regarding complaints.”—EV010
Reinforcement	The way I am paid (more visits = more money) discourages me from taking the time to have a conversation with patient about deprescribing and appropriate alternatives (2)	“And so when, you know when doctors are paid fee for service, that motivates them to do that and then that makes it much more difficult for people who are trying to practice in a responsible, ethical way in order to be able to make a living.”–EV009 “So, yeah. Like, but, yeah, that, so, it just takes so much time to educate. And I love doing it, I love to talk, as you can tell I love to talk to people, that’s not a problem. But, I don’t get paid well to talk to people. Which sucks. Cause I wish I did. But I don’t. I get paid by how many people I see. And how fast I see them. And our current fee for service tier model is a huge barrier to providing care complexly. Like, if we went to a blended capitation where like I met certain goals or I was able to do certain things based on like whatever, it would be way better. Like our current fee doesn’t allow for me to do the education and to do the I guess the leg work I need to do to either deprescribe or explain why I am not prescribing. So if you don’t pay me appropriately now that’s okay I’ll just make that sacrifice but there’s doctors who can’t and they won’t. Because they can’t pay their bills. Like they just can’t do it.” -EV010

The analysis also revealed five enablers for opioid deprescription which we describe below and presented in no particular order of significance. In our estimation, each of these areas is worthy of consideration for future intervention planning. The enablers and supporting quotations from interview participants can be found in Table 2. They were related to ten TDF domains (beliefs about consequences, goals, emotion, social professional role and identity, social influences, behavioral regulation, environmental context and resources, memory attention and decision-making processes, reinforcement, and skills).

**Table 2:** Enablers to opioid deprescription as reported by rural NL family physicians.

**Enabler 1: Understanding the problems with overprescription of opioids and sharing common views on this issue and the importance of deprescription with colleagues helps physicians to deprescribe**		
TDF Domain	Belief statement (n)	Supporting quotations
Beliefs about consequences	Deprescribing opioids will prevent negative patient/public consequences such as issues related to tolerance, addiction to prescription opioids and overdose (3)	And the risks um, being numerous things not just addiction but the hyperalgesia is that what you said? “Yes. Hyperalgesia, addiction, tolerance…overdose.”—EV001
	If I decrease my patients opioid use, it may reduce their pain (1)	“If they are taking the medicine for pain, is likely worse in the long run from with secondary effects of opioid hyperalgesia than it would be if they weren’t taking it. That’s sometimes a different reality versus their own expectation. In the long-term decreasing their opioid use will hopefully have the effect of diminishing pain and reliance on medicine for it.”—EV008
Emotion	I worry that my patients may be misusing their opioid medications—either selling them or have become addicted (1)	“So those were the people that I found more concerning, because I’m wondering if they’re selling that product on the street or I’m worried that they are addicted to their medication.”—EV005
	Opioid overprescription causes stress for physicians	“The opioid issue honestly, because it’s-it’s a huge issue, causes a lot of stress, I think to a lot of physicians” EV009
Goals	I want to transition patients to a less harmful, substitute medication (3)	“But I mean the overall goal would be to hopefully get them off opioids or get them to a minimum dosage if you can get them, you could use that strategy if you’re trying to just get them on an overall lower dose.”—EV002 “And then I tell my patients that my goal for them would be, ah to wean you off these medications, try new ones, if I am going take over your care.”—EV006
Social influences	My colleagues’ opinions influence my decision to deprescribe (2)	“And we all see each other’s charts, so if I saw you know a prescription for 400 you know, you know ah morphine 50s, you know I’d, everybody, I would see that and be like that’s a little bit odd. You know we get to sorta see each other’s charts so… there’s an element of ah your peers will know everything you do anyway…. If other physicians think I’m doing like, don’t like the plan he comes and talks to me and says ‘okay that’s what I think, what do you think?’ and then we make up a plan together.”—EV003
Social professional role and identify	My colleagues and I agree on how to manage opioid deprescription (4)	“R: And it really is dependent on physicians. So it is, here I think we don’t have, like we’ve made, we’ve made an agreement cause Percocet is a problem, or had been, we just said we don’t prescribe it.”- EV003 “In small communities like our community you kind of need everybody to be on the same page. It doesn’t really work if somebody’s trying to aggressively deprescribe and others are more free with their deprescribing. I think in our community everybody is somewhat on the same page in that regard.”- EV007
**Enabler 2: Physicians believe access to other healthcare professionals, community-based resources, and clinical tools helps/will help them to deprescribe opioids**		
TDF Domain	Belief statement (n)	Supporting quotations
Behavioral regulation	Access to other healthcare professionals and community-based resources makes/would make deprescribing opioids easier (e.g. physiotherapy, massage therapy) (9)	“Physiotherapy, massage, mental health supports, hypnosis, acupuncture [would support deprescription].”—EV009 “A full addiction team would be [useful] and I’m not sure if they exist…But you would have your…psychiatrist [or] a physician who is trained in narcotics or has extra training in de-prescribing…, your addictions counselor…[such as] a social worker or a psychologist, and then a clinical pharmacist would be great as well. So, the three of them. The pharmacist can explain the actual drug itself and what it does and what we don’t want it to do and decreasing it what you’ll, what the side effects will be and if we don’t change it, what the outcomes will be. So, the negative outcomes if you continue to stay on this. The psychologist would be more-so around the therapy and counseling of that, and you know its going to be hard coming off of it, but you know, a year down the road when you’re off of this, you’re going to feel a lot better. And then the physician is kind of the person, well obviously the one who prescribes it but kind of pieces all that together and kind of reiterates the other two. Because if you have three people telling you the same thing, you’re more inclined to believe it and do it.”—EV004
	Improved clinical tools (e.g., consult, tool kits, patient education resources) would help me deprescribe opioids (3)	“Something you can access quickly in like 5 minutes find the answer that you need so you don’t have to read the whole thing or take the whole course at once. That’s what rural physicians need. They need quick point-of-care tool kits to use on the spot.”—EV007 “I need an e-consult or something. But they [pain specialists] do not participate in the e-consult yet. If the pain people participate in the e-consult that would be great I would just send a letter saying my ‘patient is on this and this and that and that’s his condition, that’s his CT or MRI, I need to de-prescribe are you okay with this plan’. If he says yes, I have the blessing of a specialist then it’s going to protect [me].”—EV001
Environmental context and resources	I have access to support from other healthcare providers and/or community-based resources that helps me to deprescribe opioids (3)	“I actually touch base with the local pharmacy [and] I find the clinical pharmacists in the hospital, which I work in a hospital on a regular basis, are quite helpful in like the tapering of doses or the changeover in medications. That’s who I often call a lot of the time to find a proper regimen of how to do things.”- EV004 “We do have an anesthesiologist here who does do a chronic pain clinic so that has been a help.”—EV005
**Enabler 3: Physicians believe that approaching deprescription systematically helps them to deprescribe**		
TDF Domain	Belief statement (n)	Supporting quotations
Memory, attention, decision-making processes	I consider patient related factors (e.g., exam/imaging findings, type of pain, their history with a medication, a patient’s total needs) when deprescribing opioids (4)	“Because a lot of patients will just say, well I’m on it because I’ve always been on it and I need it for my back. So [for] a lot of them I went back to square one and did another thorough workup. So, imaging of their back if it hadn’t been done in a long time, [referring] them to physio. We do have an anesthesiologist here who does do a chronic pain clinic so that has been a help. And then looking at other medications…getting them on regular Tylenol if appropriate…”—EV005
	I practice evidence-based medicine and follow guidelines on prescribing/deprescribing opioids (2)	“I then go on and talk about how I’m a recent grad and I practice evidence-based medicine, [advising] for non-cancer pain, the recommendations that opioids aren’t the best thing, that kind of thing.”—EV006
	Setting clear expectations and regularly/frequently following up with patients who are in the process of stopping an opioid medication helps me to deprescribe successfully (1)	“If you [follow-up] regularly, often those appointments don’t need to be as long, like it’s just ‘okay, I’m checking in, how are you doing with your withdrawal, what’s your side effects like’… you know, that kind of thing. Whereas if you wait too long, people often give up because there will be some withdrawal of course. Because I’ve told people ‘you’re going to have these symptoms in the first two or three weeks. Let me see you at the end of two weeks so I can coach you through it’.”—EV004
**Enabler 4: Previous experience successfully deprescribing patients encourages physicians to continue deprescribing opioids**		
TDF Domain	Belief statement (n)	Supporting quotations
Reinforcement	I have successfully helped my patients to stop taking opioids or decrease their doses (2)	“Yes, I’ve been successful getting some people off or at a decreased dose.”—EV004 “Some patients were actually very reasonable [and] some patients didn’t even realize that their medication was addictive or harmful. Some of them were more than happy to come up with a plan to get them off of it.”–EV005
**Enabler 5: Practicing rurally helps physicians to deprescribe opioids**		
TDF domain	Belief statement (n)	Supporting quotations
Skills	Being in a rural area has allowed me to develop the skills necessary for deprescribing opioids (1)	And I think there’s a big difference too between rural and urban practice. Rurally, I think we just get really comfortable with just doing things, because we don’t have access and we just learn it and we do it. I find the [urban]docs…they’re great, it’s just where there’s more of them and they have easier access to specialists like their patients don’t have to travel mine are three hours. My experience as a practicing rural physician makes be better able to handle opioid deprescription.—EV010
Environmental context and resources	Being in a rural area can prevent factors that complicate deprescribing such as double-doctoring (1)	Is there are anything specific to your geographic location that would be a barrier? “Actually no. Actually, in my geographic location I think it’s a bonus that I work in a small place so that issues with double doctoring and going to different pharmacists is not an issue in our practice because all the patients have only one paper chart which everyone of us has access to.”—EV001

### Barriers to opioid deprescription

#### 1. Physicians lack knowledge, skills, and experience related to deprescribing

Participants reported that their education did not comprehensively cover chronic pain management (knowledge). They also reported lacking knowledge and experience in how to approach deprescribing, helping patients to overcome barriers during the weaning process and the use of alternative treatments (e.g., the use of cannabis as a substitute medication or skills related to administering a nerve block (knowledge, skills). Relatedly, younger physicians who are new to practice feel that their lack of experience as a newer physician makes patients reluctant to initiate deprescription—particularly when their previous, more experienced provider had been prescribing them opioids for many years (social professional role and identity).

#### 2. Physicians are uncomfortable initiating deprescription and fear it may do more harm than good

Participants reported difficulty deprescribing opioids, especially for patients with more complex issues such as multiple comorbidities and social issues (beliefs about capabilities) describing it as a stressful and anxiety-provoking part of their work (emotion). Initiating the conversation with patients who had been taking opioids for many years was reported to be particularly challenging (beliefs about capabilities). Participants believe that deprescription could lead to potential harm (beliefs about consequences). They fear that initiating these conversations might damage their therapeutic relationship (emotion) and reported past experiences in which patients did not return to their clinics after they initiated the conversation about deprescription or would not follow up with them after initiating the deprescription process (reinforcement). They are concerned that deprescribing opioids may negatively affect their patient’s quality of life or lead patients to search for opioids elsewhere where they won’t be properly managed (beliefs about consequences). Physicians also expressed concern that patients may experience severe symptoms of withdrawal if they were to deprescribe opioids (beliefs about consequences).

#### 3. Physicians are pressured by patients to continue prescribing opioids

All participants reported being pressured by patients or their family members to continue prescribing opioids (social influences). Patient reactions to deprescription (e.g., fears about their ability function without opioids and subsequent reluctance to try alternative strategies) influence physicians’ ability to deprescribe—particularly when patients become argumentative or aggressive (social influences). Participants also reported that inheriting patients who are already taking opioids makes deprescribing more difficult (environmental context and resources) as those medicines were prescribed by another doctor with whom the patient had as established relationship. This places the new physician in the difficult position of trying to move the patient off the medication they believe they need to function which is in direct opposition to how their previous, trusted physician had been treating their pain.

#### 4. Physicians lack foundational support required to deprescribe opioids

Participants reported a lack of community-based supports or alternative care providers to which they could refer patients to assist with the process of deprescribing (environmental context and resources). For example, they don’t have access to physiotherapy, massage therapy, counselling supports, or specialized pain clinics for patients in general and for patients that require publicly funded services the situation is even more dire (because they do not have the resources to cover the costs of private treatment programs and substitute medications). Some physicians also noted difficulty in accessing pharmacies to refer patients to for methadone and suboxone clinics (environmental context and resources). Participants also reported lack of access to educational resources (e.g., public presentations or campaigns about the side effects of prescription opioids). Participants also noted the lack of access to recreational facilities as being a barrier (environmental context and resources).

Poor mechanisms of communication among providers point to another area which leads physicians to feel unsupported in efforts to deprescribe (environmental context and resources). Participants reported that their lack of communication with other types of providers leaves them unaware of how services offered by other providers could play a role in or affect their treatment plan.

Although mentioned less frequently, participants raised two additional issues that contribute to a lack of support to deprescribe. First, a participant explained that fear of censure from the college of physicians and surgeons makes them more reluctant to deprescribe opioids (social influences). They worry that a conflict with a patient regarding deprescription will lead to a complaint to the college of physicians and surgeons where they will be summarily suspended while a review of the case is ongoing. A second participant reported the fee-for-service (FFS) remuneration model (which rewards physicians for seeing the most patients possible) combined with a lack of billing codes to compensate for longer, education-based appointments, fails to support deprescription by discouraging physicians from taking the time to have conversations about deprescription and appropriate alternatives with their patients (reinforcement).

### Enablers for opioid description

#### 1. Understanding the problems with overprescription of opioids and sharing common views on this issue and the importance of deprescription with colleagues helps physicians to deprescribe

Participants believe that if they don’t deprescribe, their patients may experience many negative consequences including developing drug tolerance leading to ever-increasing dosages, addiction to their medications, overdose, or development of hyperalgesia (beliefs about consequences). One participant noted that the overuse of opioids in general is an issue that causes a lot of stress for many physicians (emotion) and felt it is a significant problem that needs to be addressed (goals). Similarly, physicians noted that they feel anxious since they know the pain their patients are experiencing is simply being masked by the opioids they are taking (emotion). Their goal is to engage in deprescription by either taking their patients off opioids altogether, transitioning them to a less harmful, substitute medication or reducing their opioid dosage so they are not as dependent on the drug (goals).

Participants reported agreeing with their colleagues on how to manage opioid deprescription and felt that agreement was important for successfully deprescribing opioids (social professional role and identify). One participant noted that they will often come up with a plan to approach deprescription in consultation with their colleagues (social influences). They noted that the opinion of their colleagues influenced their deprescription practices. Since their colleagues would be able to view their charts, they would know if they continued to prescribe opioids inappropriately thereby motivating them to follow appropriate practices (social influences).

#### 2. Physicians believe access to other healthcare professionals, community-based resources, and clinical tools helps/will help them to deprescribe opioids

Participants reported that access to other healthcare professionals (particularly those trained specifically in deprescribing) and community-based resources (e.g., addictions counsellors, massage therapists, physiotherapy, pharmacists, psychiatrists, pain clinics and methadone clinics) makes deprescribing opioids easier (behavioral regulation). For example, some participants reported that working with a local pharmacist was helpful in explaining the effects of the drugs to patients and how the deprescription process would work (environmental context and resources). Another reported that their access to an anesthesiologist who runs a pain clinic was helpful, especially for steroid injections in areas that family doctors do not typically inject (environmental context and resources).

Physicians also reported that improved access to clinical tools would help them to deprescribe. This includes quick point-of-care tool kits for deprescribing opioids, access to e-consult for pain specialists, educational resources for patients, and multidisciplinary programs (behavioral regulation).

#### 3. Physicians believe that approaching deprescription systematically helps them to deprescribe

In terms of approaches to deprescription, participants reported that considering several patient-related factors such as exam or imaging findings, the type of pain the patient is experiencing, the patient’s history with a medication, and the patient’s overall needs helps them navigate the deprescription process and determine the most appropriate course of treatment. They may also return to square one, completing a thorough work-up to assist them in deciding how to approach the process (memory, attention, decision-making processes). One participant mentioned that setting clear expectations and regularly following up with their patients who are in the process of stopping an opioid prescription helps to facilitate the deprescription process as they can help coach their patient through the symptoms that may accompany the weaning process (memory, attention, and decision-making processes). Another reported that they approach deprescription by explaining that evidence does not support the use of opioids for CNCP and that they are a physician who practices evidence-based medicine (memory, attention, and decision-making processes).

#### 4. Previous experience successfully deprescribing patients encourages physicians to continue deprescribing opioids

Two physicians described previous success stories with deprescription where they were able to help their patients complete the weaning process or transition to a decreased dose. They reported that some patients were not aware that their medications were causing them harm and were agreeable to the process once they realized the benefits (reinforcement).

#### 5. Practicing rurally helps physicians to deprescribe opioids

One physician reported that practicing in a rural area has allowed them to develop the necessary skills for deprescribing opioids, explaining that since rural physicians often don’t have easy access to specialists and other resources as urban centers, they have had to become more comfortable with the deprescription process on their own (skills). Another participant reported that being in a rural area can prevent factors that complicate deprescribing, explaining that working in a small, rural community can help prevent issues such as double-doctoring or visiting multiple pharmacies (environmental context and resources).

## Discussion

This study used the TDF to conduct a theory-informed analysis of barriers and enablers to opioid deprescription among rural family doctor’s practicing in NL, Canada. Briefly, our analysis revealed four barriers and five enablers related to opioid deprescription in rural primary care. Many of the barriers and enablers noted in our study are applicable to family physicians practicing in both rural and urban contexts. In our estimation, however, we believe the most important consideration for rural settings is the need for foundational supports for deprescription. Rural physicians and patients face poor access in a variety of areas but it is particularly challenging for complicated issues like deprescription. Participants in our study struggle with poor access to alternative therapies to help with pain management including physiotherapy, massage therapy, counselling supports, or specialized pain clinics. Several participants also reported poor access to pharmacies that offer methadone and suboxone clinics. Poor mechanisms of communication among providers point to another area which leads physicians to feel unsupported in efforts to deprescribe. The importance of these supports is further highlighted by the fact that several of our participants reported that when these types of factors are available, they act as facilitators for deprescription. For example, one participant described how working with local pharmacists who could provide advice on appropriate dosing for drug tapering plans helped them deprescribe.

### Findings relative to previous research

Our findings were largely supported by the literature investigating opioid deprescription barriers and facilitators—particularly with regard to barriers. For example, all the barriers found in this study were previously reported in other papers [16,26–31]. Facilitators were less commonly reported in the literature but were also largely similar to those found in our study [16,27,28,31]. Unlike other studies, however, participants in our study noted two additional facilitators not previously reported. Unique facilitators noted in our study included previous experience successfully deprescribing patients, the skills developed through their experience practicing rural family medicine, and the nature of living in a rural community which they felt limited the ability of patients to engage in behaviors such as double-doctoring and visiting multiple pharmacies which can complicate the deprescription process.

### Implications for practice

The barriers and enablers reported in this study are nuanced and often relate to multiple TDF domains. This means that addressing them will require an equally nuanced approach. For example, in order to address known barriers an intervention would need to include upskilling to provide general knowledge about deprescribing but also training on how to manage emotionally charged conversations about deprescription. It should also inform clinicians’ understanding of patients’ expectations about pain management and their understanding of opioid treatment and provide patient education materials and improved access to community-based patient supports. From a practical perspective, parts of this intervention would be easy to implement such as providing basic upskilling on tapering protocols. However, other parts would require more in-depth provider training and changes to services to provide additional supports for patients. These latter elements are more challenging to implement. For example, providing in-depth training isn’t always a feasible solution as it is resource intensive—it is costly and takes a lot of time. Even if this kind of provider training was implemented, changes to billing structures may be required to support physicians to spend the extra time necessary for deprescription by compensating them for the time spent on these issues. Otherwise, competing pressures for time and income would likely undermine any potential success to be gained from other interventions. Most importantly, mechanisms to support coordination and access to community-based chronic pain services is paramount.

### Future research

Our results align closely with findings from the larger literature base including several moderate to high confidence findings of a TDF-based systematic review of barriers (and enablers) to opioid deprescription. As a result, and given the pressing need to address the opioid crisis, we believe there is sufficient information available to inform the selection or development of an intervention to improve opioid deprescription. In parallel, future research could also explore prevalence of the physician-identified barriers to deprescription in the province and elsewhere as well as additional barriers with larger samples of physicians using theory-informed questionnaires.

As physicians reported multiple barriers mapped to a number of TDF domains, we believe a multicomponent intervention using multiple theory-based behavior change strategies is warranted. Michie et al [32–34] have developed a set of resources to complement the TDF. The Behavior Change Wheel [33] can be used by researchers to guide the design of behavior change interventions. The Behavior Change Technique Taxonomy [32] is an extensive list of 93 behaviour change techniques that can be used to form the active components of an intervention. Finally, the Theory and Techniques tool [34] helps researchers to map behavior change techniques with TDF domains in order to choose the most relevant techniques for the barriers they have identified. For maximum impact, future research should focus on developing and testing multicomponent interventions that focus on both physicians and patients by addressing both physician-identified barriers and patient-identified needs related to opioid deprescription (e.g., what they need to support them through the process, and how they wish to communicate with their physician and/or medical team. However, before developing bespoke approaches, researchers should examine the feasibility of existing interventions that have been developed to tackle these issues. Unfortunately, there are few well-designed, randomized trials investigating physician-focused opioid deprescription. We found only one study that improved adherence to prescribing guidelines that involved education plus decision-support tools [35]. Trials of patient-focused interventions (e.g., iWOTCH [36]), on the other hand, are far more common.

### Strengths and limitations

This study used a theoretical framework to guide our analysis of physician behaviors related to opioid deprescription as recommended by many health and research organization [37–40] for investigations of behavior change. We used the Atkins et al [23] guide to direct our analysis, thereby allowing us to produce results that can be used to develop a theory-based intervention that includes evidence- and empirically-based strategies to tackle identified barriers to opioid deprescription. We also applied rigorous methods (including extensive coder training, double coding of all transcripts, and oversight of the analysis by senior researchers experienced in application of the TDF for assessment of barriers and enablers to behavior change) and used the Consolidated Criteria for Reporting Qualitative Research (COREQ) 32-item checklist [41] to guide our methods and reporting as a method of reducing reporting bias.

Despite these strengths, several limitations of our study may have led to missed information. First, we did not use a TDF-informed interview guide which is typically designed to access information related to every domain. Study interviewers were also not experts in the content area (opioid deprescription). Additionally, we did not provide participants with an opportunity to review their transcripts. Since we had the audio-recordings against which we would check the accuracy of the transcription, the team felt it would be unnecessarily burdensome to request further time of these physicians to review their transcripts. However, we realize this precluded participants’ ability to clarify and reflect on their responses. Finally, although we conducted ten interviews and informally assessed data saturation, we did not carry out the formal data saturation assessment that was originally planned and described in the method section of this manuscript; it is possible that additional interviews could have given rise to novel information. That being said, we were able to recruit physicians with a range of experience from one to ten years in rural practice from each of the province’s regional health authorities, allowing us to access a reasonably diverse array of perspectives and our results were consistent with other work in this area.

## Conclusion

Opioid dependence and overprescription continues to be a problem for our health system. Deprescription is necessary but this and other studies [16,26–31] show that it is challenging for family physicians. Physicians are keenly aware of the importance of preserving the physician-patient therapeutic relationship and open and clear communication about opioid medications and deprescription but feel unprepared to manage this in the face of difficult issues surrounding deprescription. They also feel unprepared to deal with deprescription effectively without access to other resources, healthcare professionals, patient education materials and time. Rural physicians would benefit most from added foundational supports for deprescription.

## Supporting information

S1 Appendix Interview guide. (DOCX)
