# Supplementary material for: Barriers and enablers to opioid deprescription: A qualitative study
Source: PLoS One. 2025 Jan 7;20(1):e0316730. doi: 10.1371/journal.pone.0316730 (PMC11706500; doi:10.1371/journal.pone.0316730)
Supplement: S1 Appendix — (DOCX) [file pone.0316730.s001.docx]

**Interview guide**

Hi, my name is [interviewer name] and I am conducting research on barriers to opioid deprescription in rural Newfoundland. I would like to ask you a few questions on this topic and the process should take about 30 minutes.

1. How long have you been in rural practice? How long have you been prescribing opioids to patients?
2. In your experience, what have been the biggest barriers when discontinuing opioid therapy with your patients? Please explain.
3. Are there any supports or resources available to you with respect to opioid deprescription? Please explain.
4. Are there any barriers you see as specific to rural practice or Newfoundland? Please explain.
5. Do you have anything else to add or any questions for me?

Thank you for your input and your time. I will forward my results of this study when they are complete.
